# Cerebral blood flow in the paracentral lobule is associated with poor subjective sleep quality among patients with a history of methadone maintenance treatment

**DOI:** 10.3389/fneur.2024.1400810

**Published:** 2024-08-08

**Authors:** Jiaxue Sun, Yi Lu, Deshenyue Kong, Wenhua Lin, Jinze Du, Guangqing Wang, Xingfeng Ma, Congbin Li, Kunhua Wang, Mei Zhu, Yu Xu

**Affiliations:** ^1^First Affiliated Hospital of Kunming Medical University, Kunming Medical University, Kunming, China; ^2^Yunnan Technological Innovation Center of Drug Addiction Medicine, Yunnan University, Kunming, China; ^3^Drug Rehabilitation Administration of Yunnan Province, Kunming, China

**Keywords:** arterial spin labeling, cerebral blood flow (CBF), methadone maintenance treatment (MMT), paracentral lobule, sleep disturbance

## Abstract

**Introduction:**

Sleep disorders are prevalent and significant among individuals receiving methadone maintenance treatment (MMT), adversely affecting their quality of life and treatment adherence. While cerebral blood flow (CBF) plays a crucial role in the development of various diseases, its relationship with sleep disorders remains uncertain. This observational study focuses on possible correlations between CBF and poor subjective sleep quality in MMT patients.

**Methods:**

A total of 75 participants with a history of MMT were recruited and assessed using pseudo-continuous arterial spin labeling magnetic resonance imaging to determine CBF. A LAASO regression model was employed to identify the region of interest (ROI) most associated with sleep disturbance. The association between the CBF of the ROI and the Pittsburgh Sleep Quality Index (PSQI) was examined using regression analyses. Age, gender, BMI, history of hypertension, diabetes, hyperlipidemia, and methadone withdrawal were included as covariates.

**Results:**

Among MMT patients with poor subjective sleep quality, significantly higher CBF was observed in the right paracentral lobule (56.1057 ± 11.1624 ml/100 g/min, *p* = 0.044), right cerebelum_3 (56.6723 ± 15.3139 ml/100 g/min, *p* = 0.026), right caudate nucleus (48.9168 ± 6.9910 ml/100 g/min, *p* = 0.009), and left caudate nucleus (47.6207 ± 6.1374 ml/100 g/min, p = 0.006). Furthermore, a positive correlation was found between CBF in the right paracentral lobule and the total PSQI score (β = 0.1135, *p* = 0.0323), with the association remaining significant even after adjustment for covariates (β = 0.1276, *p* = 0.0405).

**Conclusion:**

MMT patients with poor subjective sleep quality exhibited significantly altered CBF in multiple brain regions. The association between increased CBF in the right paracentral lobule and subjective sleep quality in MMT patients could be crucial in understanding sleep disorders in individuals undergoing MMT.

**Clinical trial registration:**

https://www.chictr.org.cn/, identifier: ChiCTR2100051931.

## Introduction

Opioid addiction remains a pervasive and challenging public health issue, characterized by high levels of abuse and associated mortality (1). Opioid-related deaths continue to increase at unprecedented rates in many regions of the world (2). Various treatment options have been explored and implemented worldwide, with opioid substitution therapy having emerged as a cornerstone in managing heroin addiction (3). Methadone is the most widely used medication in China (4) for reducing illicit drug use, minimizing withdrawal symptoms, and promoting long-term recovery. It plays a crucial role in harm reduction and treatment (5). However, numerous challenges in methadone maintenance treatment (MMT) still affect patient quality of life and long-term treatment outcomes (6, 7).

Sleep disturbances, such as insomnia, hypersomnia, and fragmented sleep patterns, are commonly reported among individuals with opioid dependence (8–11), further exacerbating the challenges of withdrawal and recovery. It is also a common problem self-reported by MMT patients (10, 11) and can impact patients' compliance (12) and long-term effectiveness. Moreover, recent studies have highlighted the complex relationship between addiction, withdrawal symptoms, and sleep disturbance. Sleep disturbance not only exacerbates the challenges of withdrawal but also contributes to a cycle of vulnerability (13), as poor sleep quality is linked to an increased risk of relapse (14). The psychological issues and changes in brain function resulting from prolonged sleep disturbance may increase the likelihood of relapse and negative outcomes (15). Addressing sleep disturbance not only improves patients' quality of life adherence to therapy and promotes long-term recovery but also disrupts the detrimental cycle of sleep disturbance and addiction.

A recent study has begun to report on the impact of regional cerebral blood flow (CBF) in neuropsychiatric disorders (16). Acute effects of opioid receptor agonists increase CBF (17), whereas long-term heroin dependence results in a significant decrease in regional CBF (18), which can be used as an imaging marker to distinguish drug users (19). Patients with sleep disturbance or sleep deprivation show significant changes in CBF (20–22), demonstrating the complexity of CBF's role. The paracentral lobule, a brain region implicated in motor control and cognitive functions (23), plays a crucial role in shaping the neural landscape of addiction (24) and sleep disturbance (25). However, some studies have reported conflicting results, indicating the need for further research (26–28). The relationship between CBF in the paracentral lobule and sleep disturbance in MMT patients is poorly understood (29).

In this context, we explored the relationship between regional CBF and sleep disturbance in MMT patients. Our analysis revealed an independent association between paracentral lobular CBF and subjective sleep quality in MMT patients. This study provides insights into the mechanisms of substance use disorder (SUD) and sleep disturbances. Understanding the neurobiological mechanism of sleep disturbances in MMT patients can inform the development of strategies to address the multifaceted challenges posed by SUD and advance evidence-based interventions for its management.

## Materials and methods

### Participants

A cohort of 75 right-handed participants was recruited by the researchers at the Yunnan Technological Innovation Center of Drug Addiction Medicine and the First Affiliated Hospital of Kunming Medical University from outpatient rehabilitation clinics and local communities. The inclusion criteria were as follows: (a) currently or previously meeting the Diagnostic and Statistical Manual of Mental Disorders-V for SUD, (b) currently or previously enrolled in an MMT program, and (c) having a negative urine test for illicit drugs. All patients received an MRI scan after providing written informed consent. The exclusion criteria included (a) difficulty with reading and writing and (b) having cardiac pacemakers, metallic ocular fragments, or metallic implants. Subjective sleep quality was assessed using the Pittsburgh Sleep Quality Index questionnaire (PSQI), a widely used questionnaire to assess sleep quality over the past month with seven components for seven specific sleep features. MMT history was determined through retrospective interviews. MMT withdrawal (MMT-W) was defined as discontinuation of methadone for more than 3 months.

This study was approved by the Ethical Committee of the Clinical Research Ethics Committee at the First Affiliated Hospital of Kunming Medical University (2021-l-2). All participants provided written informed consent for the sample, clinical data collection, and subsequent analyses before participating in the study. The investigation was conducted in accordance with the latest version of the Declaration of Helsinki. This clinical trial was registered in the Chinese Clinical Trial Registry with the code ChiCTR2100051931.

### MRI data acquisition

Image acquisition was performed on a 3T MR scanner (Discovery 750w, GE Healthcare, Milwaukee, WI, USA) with a 32-channel head coil. Arterial spin labeling (ASL) was performed using a three-dimensional pseudo-continuous arterial spin labeling (3D pCASL) sequence with the following parameters: axial acquisition, TR = 5070ms, TE = 11.5ms, FOV = 240 × 240 mm^2^, acquisition matrix = 128 × 128, slice thickness = 3mm, slice number = 50, post-labeling delay = 2,025 ms, and scan time = 4 min 54 s.

### Preprocessing of MR imaging data

Data processing was conducted using CereFlow software (Anying Technology (Beijing)) with the following steps: (1) Calculation of CBF from the GE scanner's ASL's perfusion-weighted (PW) image and proton density (PD) image using the standard simple compartment model with an assumption that the arterial transit time (ATT) is equivalent to the post-labeling delay (PLD); (2) normalization of the PD image to the Montreal Neurological Institute (MNI) template; (3) warping of the CBF image into the MNI space using the forward transformation matrix derived from the PD image; and (4) extraction of the regional CBF by the Automatic Anatomical Labeling (AAL) and Brainnetome (BN) atlases. The ROIs selected for further analysis included the frontal lobe, the mesolimbic system, and the cerebellum.

### Statistical analysis

Data were analyzed using EmpowerStats (http://www.empowerstats.com). Differences between the groups were assessed using either the Student's t-test or Welch's t-test. LASSO regression and generalized linear regression were conducted using EmpowerStats (http://www.empowerstats.com) and R (4.2.0). Significant differences are indicated in the figures by ^*^p < 0.05, ^**^p < 0.01, and ^***^p < 0.001. Notably, nearly significant differences (0.05 < *p* < 0.1) are indicated in the figures. We assessed normality using the Pearson Chi-Square test.

## Results

### Participants

Demographic and clinical characteristics of the MMT patient are presented and compared in Table 1. The sample includes 51 MMT patients with poor subjective sleep quality and 24 without. The groups were formed based on the total PSQI score (poor subjective sleep quality: total PSQI score > 5). The total PSQI score and each PSQI subscore showed significant differences. However, there were no significant differences between the two groups in terms of age, gender, BMI, education level, smoking status, drinking status, history of chronic diseases (diabetes, hypertension, and hyperlipidemia), and methadone withdrawal status.

**Table 1:** General characteristics of the study population.

	**Poor subjective sleep quality**		
	**No**	**Yes**	***p-value***
N	24 (32.00%)	51 (68.00%)	
Age	52.583 ± 4.960	51.196 ± 6.148	0.337
Height (cm)	166.375 ± 7.252	165.620 ± 6.543	0.655
Weight (kg)	62.458 ± 11.666	62.890 ± 9.999	0.87
BMI (kg/m^2^)	22.500 ± 3.355	22.858 ± 2.937	0.64
**Gender**			0.275
Men	9 (37.5000%)	26 (50.9804%)	
Women	15 (62.5000%)	25 (49.0196%)	
**Age**			0.287
≤ 40	1 (4.167%)	2 (3.922%)	
41–50	4 (16.667%)	16 (31.373%)	
51–60	19 (79.167%)	30 (58.824%)	
>60	0 (0.000%)	3 (5.882%)	
**Education level**			0.302
Elementary or less	4 (16.667%)	8 (16.327%)	
Junior middle school	13 (54.167%)	18 (36.735%)	
High school and beyond	7 (29.167%)	23 (46.939%)	
**History of diabetes**			0.879
No	19 (95.000%)	46 (95.833%)	
Yes	1 (5.000%)	2 (4.167%)	
**History of hypertension**			0.626
No	17 (80.952%)	34 (75.556%)	
Yes	4 (19.048%)	11 (24.444%)	
**History of hyperlipidemia**			0.092
No	23 (95.833%)	39 (81.250%)	
Yes	1 (4.167%)	9 (18.750%)	
**Smoking status**			0.325
No	0 (0.000%)	2 (3.922%)	
Yes	24 (100.000%)	49 (96.078%)	
**Drinking status**			0.404
No	14 (58.333%)	23 (47.917%)	
Yes	10 (41.667%)	25 (52.083%)	
**Methadone withdrawal**			
No	7 (29.167%)	23 (45.098%)	0.189
Yes	17 (70.833%)	28 (54.902%)	
**Total PSQI**			< 0.001
**[Median (min-max)]**	4 (1.0000–5.0000)	9 (6.0000–21.0000)	
**Subjective sleep quality factor**			< 0.001
Very good	13 (54.1667%)	4 (7.8431%)	
Fairly good	10 (41.6667%)	13 (25.4902%)	
Fairly bad	1 (4.1667%)	21 (41.1765%)	
Very bad	0 (0.0000%)	13 (25.4902%)	
**Sleep latency factor**			< 0.001
Not during the past month	6 (25.0000%)	2 (3.9216%)	
Less than once a week	12 (50.0000%)	9 (17.6471%)	
Once or twice a week	6 (25.0000%)	18 (35.2941%)	
Three or more times a week	0 (0.0000%)	22 (43.1373%)	
**Sleep duration factor**			0.002
>7 h	18 (75.0000%)	16 (31.3725%)	
6–7 h	6 (25.0000%)	19 (37.2549%)	
5–6 h	0 (0.0000%)	10 (19.6078%)	
< 5 h	0 (0.0000%)	6 (11.7647%)	
**Habitual sleep efficiency factor**			< 0.001
≥ 85%	23 (95.8333%)	19 (37.2549%)	
75–84%	1 (4.1667%)	18 (35.2941%)	
65–74%	0 (0.0000%)	6 (11.7647%)	
< 65%	0 (0.0000%)	8 (15.6863%)	
**Sleep disturbance factor**			0.015
0	3 (12.5000%)	0 (0.0000%)	
1	18 (75.0000%)	32 (62.7451%)	
2	3 (12.5000%)	16 (31.3725%)	
3	0 (0.0000%)	3 (5.8824%)	
**Sleep medication factor**			0.044
Not during the past month	24 (100.0000%)	37 (72.5490%)	
Less than once a week	0 (0.0000%)	4 (7.8431%)	
Once or twice a week	0 (0.0000%)	4 (7.8431%)	
Three or more times a week	0 (0.0000%)	6 (11.7647%)	
**Daytime dysfunctions factor**			< 0.001
0	8 (33.3333%)	3 (5.8824%)	
1	15 (62.5000%)	18 (35.2941%)	
2	1 (4.1667%)	16 (31.3725%)	
3	0 (0.0000%)	14 (27.4510%)	

PSQI, Pittsburgh Sleep Quality Index; Fisher Exact for categorical variables with Expects < 10.

### Lasso logistic regression

**Figure 1:**
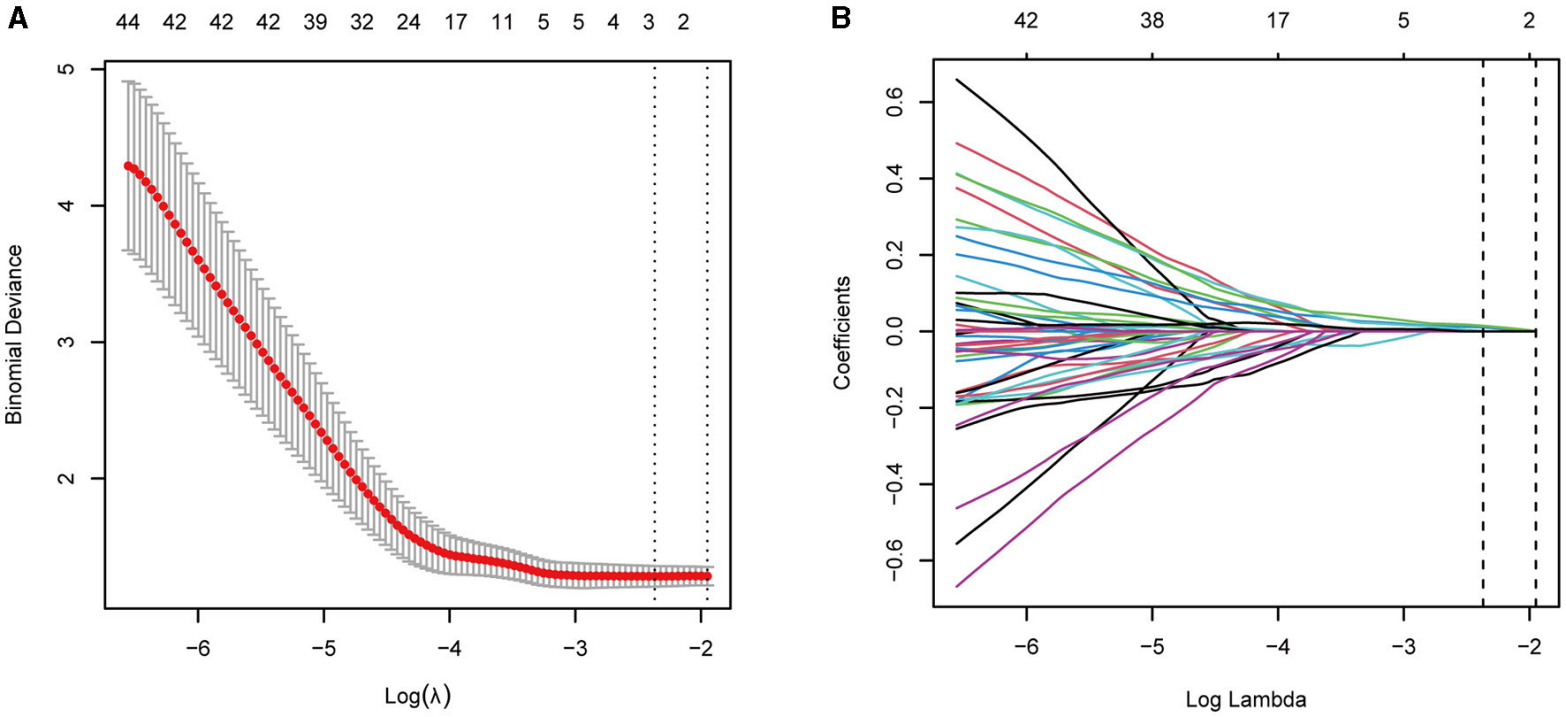
**(A, B)** LASSO regression showed log(λ) = −2.3686 when the model error was minimized, and three variables were selected for further logistic regression analysis.

Given the many variables (CBF of the cerebrum and cerebellum subregions) and relatively few cases, collinearity was expected based on empirical extrapolations. We constructed the LASSO logistic regression model to determine the variables most associated with subjective sleep quality. We utilized ten-fold cross-validation to select the penalty term, lambda(λ). Log (λ) = −2.3686 (λ = 0.0936) when the error of the model was minimized, and three variables were selected for further logistic regression analysis: right paracentral lobule, caudate nucleus, and right cerebelum_3 R (Figures 1A, B).

### The regional CBF of patients undergoing MMT

**Figure 2:**
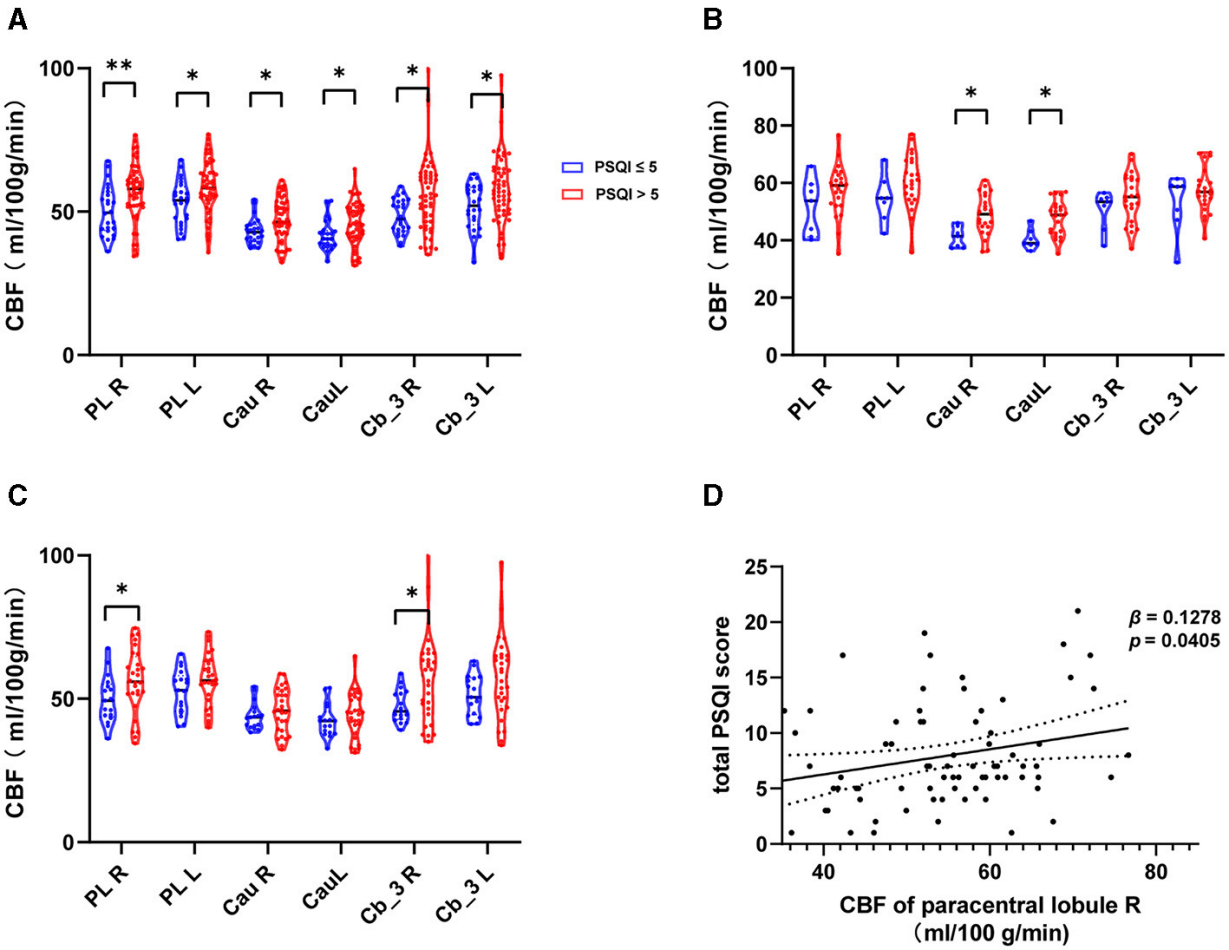
The CBF of ROIs in MMT patients with poor sleep quality. **(A–C)** CBF of ROIs in all patients, MMT patients, and MMT-W patients. **(D)** Generalized linear regression model for CBF of the right paracentral lobule and total Pittsburgh Sleep Quality Index (PSQI). PL, Paracentral LobuleR; Cau, Caudate nucleus; Cb_3, Cerebelum_3; R, Right; L, Left; MMT, methadone maintenance treatment; MMT-W, methadone withdrawal patients. *p < 0.05; **p < 0.01.

The CBF of the ROI was significantly higher in MMT patients with poor subjective sleep quality. We also compared the contralateral brain regions of the selected subregions above. The CBF of the right paracentral lobule (56.1057 ± 11.1624 ml/100 g/min, *p* = 0.044) and right cerebelum_3 (56.6723 ± 15.3139 ml/100 g/min, p = 0.026) were significantly higher only in MMT-W patients with poor subjective sleep quality. The CBF of the right caudate nucleus (48.9168 ± 6.9910 ml/100 g/min, p = 0.009), left caudate nucleus (47.6207 ± 6.1374 ml/100 g/min, p = 0.006) were significantly higher in MMT patients with poor subjective sleep quality (Figures 2A–C).

## Association between the CBF of left paracentral lobule and total Pittsburgh sleep quality index

The CBF of the right paracentral lobule positively correlated with the total PSQI (β = 0.11355, *p* = 0.032341). This remained an independent factor (β = 0.12764, *p* = 0.040545) for the total PSQI after correcting for confounding factors such as gender, age, BMI, history of hypertension, history of hyperlipidemia, history of diabetes, and MMT withdrawal status (Figure 2D, Table 2). We further analyzed the associations between CBF of the right paracentral lobule and PSQI sleep component domains using the multinomial logistic regression model. No significant association was found (Supplementary Table S1). We also found no significant associations between other regional CBF and total PSQI scores before or after correction for the confounding factors.

**Table 2:** Association between the CBF of ROIs and the total Pittsburgh Sleep Quality Index.

	**Non-adjusted**			**Adjust I**			**Adjust II**		
**CBF of ROIs**	β	**95% CI**	***p-value***	β	**95% CI**	***p-value***	β	**95% CI**	***p-value***
Paracentral lobule R	0.1135	0.0116, 0.2156	0.0323	0.1370	0.0309, 0.2430	0.0136	0.1276	0.0085, 0.2467	0.0405
Paracentral lobule L	0.1082	−0.0046, 0.2211	0.0641	0.1292	0.0111, 0.2473	0.0355	0.0917	−0.0464, 0.2297	0.1987
Caudate nucleus R	0.1074	−0.0438, 0.2586	0.1680	0.1175	−0.0352, 0.2702	0.1360	0.0767	−0.0973, 0.2506	0.3917
Caudate nucleus L	0.0552	−0.0974, 0.2078	0.4805	0.0613	−0.0930, 0.2157	0.4386	0.0541	−0.1169 0.2251	0.5377
Cerebelum_3 R	0.0619	−0.0292, 0.1529	0.1870	0.0674	−0.0247, 0.1594	0.1561	0.0667	−0.0295, 0.1629	0.1802
Cerebelum_3 L	0.0800	−0.0158, 0.1757	0.1058	0.0939	−0.0035, 0.1914	0.0631	0.0729	−0.0360, 0.1818	0.1954

Adjust I adjust for gender (men, women), age, BMI.

Adjust II adjusts for gender (men, women), age, BMI, history of hypertension, history of hyperlipidemia, history of diabetes, withdrawal of MMT.

## Discussion

The present study discusses the issue of sleep disturbance in patients receiving MMT, which is a prevalent problem in such patients (30). Sleep disorders in MMT patients are associated with poor quality of life, increased impulsivity, and higher relapse rates (31), highlighting the need for greater attention to this issue. These sleep disorders are not merely subjective experiences; objective evidence also exists, including respiratory disorders during sleep (32) and daily sleep EEG abnormalities (33). The present study explored the relationship between regional CBF and poor subjective sleep quality, as assessed using the PSQI, in MMT patients, particularly focusing on the paracentral lobule, caudate nucleus, and cerebelum_3.

This study showed that regional CBF in the identified ROIs was significantly higher in MMT patients with poor subjective sleep quality. Further subgroup analyses showed that increased rCBF was particularly notable in patients who had withdrawn from MMT. Additionally, the regional CBF of the right paracentral lobule was independently associated with PSQI scores, as determined using the generalized linear regression model.

This result suggests that methadone administration activates brain regions rich in opioid receptors, modulating their functional connectivity and leading to increased cerebral perfusion and disrupted sleep patterns. This finding aligns with previous studies indicating that the paracentral lobule is closely related to sleep disturbances (27, 34–37). However, some studies have reported opposite results, showing reduced regional CBF in areas such as the cerebellum, vermis, right hippocampus, and left parahippocampal gyrus, as well as a negative relationship with insomnia severity (28).

Differences across studies could also be due to the different study populations. For example, Xu et al. (28) studied patients with chronic insomnia disorder and comorbid major depressive disorder. The percentage of rapid eye movement (REM%) sleep duration was correlated with improvements in depressive symptoms through the regulation of CBF in the bilateral paracentral lobule (25). This result also deepens the understanding that perfusion contributes to the brain network and is related to sleep disturbances (38).

The study employed LASSO regression to identify brain regions associated with sleep quality, a widely accepted method for minimizing potential collinearity and isolating key variables. The contralateral brain regions of the selected ROI were also included. Finally, the study confirmed an independent correlation between the rCBF of the paracentral lobular and PSQI scores. Additionally, increased rCBF in the right paracentral lobule was identified in patients with Internet addiction (39), highlighting the significance of the paracentral lobules in addiction.

Both MMT and long-term sleep disturbance lead to significant changes in brain function and structural network, potentially causing abnormalities in cerebral perfusion and poor sleep quality. These findings suggest that the paracentral lobules play a crucial role in addiction and related behaviors. Further research in this area could provide novel insights into the mechanisms underlying addiction and potentially enhance treatment strategies for patients with sleep disturbances.

## Limitations of the study

Several limitations may be considered. First, the available sleep-related data are limited to the PSQI, lacking objective sleep quality evaluation tools such as polysomnography or the Athens Insomnia Scale. Although some studies have reported consistency between subjective sleep quality and objective evidence in MMT patients (32), the lack of objective evidence remains a limitation. Nevertheless, the PSQI is a widely used questionnaire in MMT patients (40, 41). Second, the percentage of subjects (97.33%) with smoking status raises the possibility of residual confounding.

Similarly, although we used multivariate regression, we cannot exclude the potential confounding effect of other variables due to the data characters. Third, the enrolled subjects were 35–65 years old, with a mean age of 51.8 ± 5.7 years. Although age was adjusted for as a covariate in the analysis, the representativeness of the conclusions still needs to be interpreted with caution. Fourth, the control group without a history of MMT was not included in our study, which limits our ability to determine whether our results are specific to patients with a history of MMT or are more generally related to poor subjective sleep quality. A subsequent study observed a correlation between CBF in the bilateral paracentral lobule and REM% in patients diagnosed with major depressive disorder (MDD) (25).

Interestingly, no significant correlation was found between brain blood flow in this region and sleep patterns in healthy individuals aged between 30 and 53 years. Despite variations in age groups and medical conditions, similar correlations were identified. Furthermore, recent research has indicated that functional connectivity in this specific brain region is linked to PSQI scores in individuals with sleep disorders (34). Consequently, it is hypothesized that changes in CBF in the paracentral lobule may be attributed to sleep disorders, although further investigation is required to confirm this.

Furthermore, the inclusion of emotional factors, such as depression, in the analysis should be considered another limitation. Finally, reverse causation cannot be excluded due to the cross-sectional design. It has been reported that long-term sleep disturbance and sleep deprivation could lead to changes in the structural and functional networks of the paracentral lobule, possibly leading to perfusion alterations, which is an important topic that deserves further exploration.

## Data availability statement

The original contributions presented in the study are included in the article/Supplementary material, further inquiries can be directed to the corresponding authors.

## Ethics statement

The studies involving humans were approved by the Ethical Committee from the Clinical Research Ethics Committee, First Affiliated Hospital of Kunming Medical University (2021-l-2). The studies were conducted in accordance with the local legislation and institutional requirements. The participants provided their written informed consent to participate in this study.

## Author contributions

JS: Writing – review & editing, Writing – original draft, Validation, Software, Methodology, Investigation, Formal analysis, Data curation, Conceptualization. YL: Investigation, Formal analysis, Data curation, Writing – review & editing, Writing – original draft, Methodology. DK: Resources, Conceptualization, Writing – original draft, Formal analysis, Data curation. WL: Software, Methodology, Writing – original draft, Resources, Formal analysis. JD: Validation, Investigation, Writing – original draft, Resources. GW: Conceptualization, Writing – original draft, Resources, Investigation. XM: Supervision, Project administration, Writing – original draft, Resources, Investigation. CL: Writing – review & editing, Funding acquisition, Conceptualization, Writing – original draft, Supervision, Resources, Project administration. KW: Methodology, Investigation, Writing – review & editing, Writing – original draft, Funding acquisition, Conceptualization. MZ: Supervision, Software, Project administration, Data curation, Writing – review & editing, Writing – original draft, Funding acquisition. YX: Writing – review & editing.
