# Supplementary material for: Cerebral blood flow in the paracentral lobule is associated with poor subjective sleep quality among patients with a history of methadone maintenance treatment
Source: Front Neurol. 2024 Aug 8;15:1400810. doi: 10.3389/fneur.2024.1400810 (PMC11338899; doi:10.3389/fneur.2024.1400810)
Supplement: Supplementary file 1 [file Table_1.DOCX]

Supplementary Material

# Supplementary Data

# Supplementary Figures and Tables

## Supplementary tables

| **Supplementary table 1. The associations between CBF in right Paracentral lobule and PSQI sleep component domains by Multinomial logistic regression model** | | | |  |
| --- | --- | --- | --- | --- |
|  | *β* | 95% CI | *p* value |  |
| Subjective sleep quality factor |  |  |  |  |
|  | 1 | ref. |  |  |
|  | 1.027 | 0.9446, 1.1166 | 0.5323 |  |
|  | 1.0576 | 0.9659, 1.1579 | 0.2263 |  |
|  | 1.0108 | 0.9169, 1.1142 | 0.8293 |  |
| Sleep latency factor |  |  |  |  |
|  | 1 | ref. |  |  |
|  | 0.9939 | 0.8780, 1.1250 | 0.9225 |  |
|  | 1.0298 | 0.9148, 1.1592 | 0.6268 |  |
|  | 1.0449 | 0.9241, 1.1814 | 0.4835 |  |
| Sleep duration factor |  |  |  |  |
|  | 1 | ref. |  |  |
|  | 1.058 | 0.9850, 1.1364 | 0.1219 |  |
|  | 1.0847 | 0.9798, 1.2008 | 0.1173 |  |
|  | 1.0516 | 0.9312, 1.1875 | 0.4173 |  |
| Habitual sleep efficiency factor |  |  |  |  |
|  | 1 | ref. |  |  |
|  | 1.076 | 0.9872, 1.1729 | 0.0956 |  |
|  | 0.9563 | 0.8485, 1.0780 | 0.4648 |  |
|  | 1.1923 | 1.0306, 1.3794 | 0.018 |  |
| Sleep disturbance factor |  |  |  |  |
|  | 1 | ref. |  |  |
|  | 1.1221 | 0.9483, 1.3276 | 0.1796 |  |
|  | 1.1098 | 0.9324, 1.3210 | 0.2411 |  |
|  | 1.075 | 0.8482, 1.3624 | 0.5496 |  |
| Sleep medication factor |  |  |  |  |
|  | 1 | ref. |  |  |
|  | 1.141 | 0.9629, 1.3522 | 0.1277 |  |
|  | 1.1438 | 0.9634, 1.3579 | 0.1251 |  |
|  | 1.0644 | 0.9206, 1.2306 | 0.3995 |  |
| Daytime dysfunctions factor |  |  |  |  |
|  | 1 | ref. |  |  |
|  | 1.0347 | 0.9307, 1.1503 | 0.5281 |  |
|  | 1.1117 | 0.9906, 1.2475 | 0.0719 |  |
|  | 1.1157 | 0.9896, 1.2580 | 0.0736 |  |
| adjust for: Gender (male， female); Age; BMI; history of hypertension; history of hyperlipidemia; history of diabetes | | | |  |

| Supplementary table 2. The CBF of ROIs in MMT patients with poor sleep quality (ml/100 g/min) | | | | | | | | | |
| --- | --- | --- | --- | --- | --- | --- | --- | --- | --- |
|  | Total | | | MMT | | | MMT-W | | |
|  | Poor subjective sleep quality | | *p* value | Poor subjective sleep quality | | *p* value | Poor subjective sleep quality | | *p* value |
|  | No | Yes |  | No | Yes |  | No | Yes |  |
| N | 24 | 51 |  | 7 | 23 |  | 17 | 28 |  |
| Paracentral lobule R | 50.3381 ± 8.6202 | 56.7261 ± 10.1099 | 0.009 | 51.6374 ± 9.9393 | 57.4813 ± 8.8484 | 0.148 | 49.8031 ± 8.2903 | 56.1057 ± 11.1624 | 0.044 |
| Paracentral lobule L | 53.1157 ± 7.7476 | 58.3887 ± 9.3599 | 0.019 | 54.4890 ± 8.2816 | 60.5673 ± 9.4335 | 0.137 | 52.5503 ± 7.7074 | 56.5991 ± 9.0754 | 0.175 |
| Caudate nucleus R | 43.1575 ± 4.5440 | 47.2147 ± 7.4724 | 0.017 | 41.1087 ± 3.6394 | 48.9168 ± 6.9910 | 0.009 | 44.0012 ± 4.7034 | 45.8165 ± 7.6865 | 0.386 |
| Caudate nucleus L | 41.7452 ± 5.1319 | 45.7662 ± 7.3065 | 0.018 | 40.4007 ± 3.5269 | 47.6207 ± 6.1374 | 0.006 | 42.2987 ± 5.6637 | 44.2430 ± 7.9267 | 0.349 |
| Cerebelum_3 R | 48.2891 ± 6.0461 | 55.7535 ± 12.6686 | 0.008 | 50.4382 ± 6.8391 | 54.6350 ± 8.6346 | 0.25 | 47.4042 ± 5.6707 | 56.6723 ± 15.3139 | 0.026 |
| Cerebelum_3 L | 51.7882 ± 7.8815 | 57.3103 ± 11.6684 | 0.039 | 52.6133 ± 10.3847 | 57.2684 ± 7.7851 | 0.21 | 51.4485 ± 6.9595 | 57.3448 ± 14.2388 | 0.096 |
| ^MMT:^ ^methadone maintenance treatment; MMT-W: methadone maintenance treatment withdrawal; R: right; L: left^ | | | | | | | | | |
